# Cuttlefish Species Authentication: Advancing Label Control through Near-Infrared Spectroscopy as Rapid, Eco-Friendly, and Robust Approach

**DOI:** 10.3390/foods12152973

**Published:** 2023-08-07

**Authors:** Sarah Currò, Stefania Balzan, Enrico Novelli, Luca Fasolato

**Affiliations:** Department of Comparative Biomedicine and Food Science, University of Padova, Agripolis, Viale dell’Università 16, 35020 Legnaro, Italy; sarah.curro@unipd.it (S.C.); enrico.novelli@unipd.it (E.N.); luca.fasolato@unipd.it (L.F.)

**Keywords:** chemometrics, support vector machine, untargeted method, food inspection, seafood

## Abstract

Accurate species identification, especially in the fishery sector, is critical for ensuring food safety, consumer protection and to prevent economic losses. In this study, a total of 93 individual frozen–thawed cuttlefish samples from four different species (*S. officinalis*, *S. bertheloti*, *S. aculeata*, and *Sepiella inermis*) were collected from two wholesale fish plants in Chioggia, Italy. Species identification was carried out by inspection through morphological features using dichotomic keys and then through near-infrared spectroscopy (NIRS) measurements. The NIRS data were collected using a handled-portable spectrophotometer, and the spectral range scanned was from 900–1680 nm. The collected spectra were processed using principal component analysis for unsupervised analysis and a support vector machine for supervised analysis to evaluate the species identification capability. The results showed that NIRS classification had a high overall accuracy of 93% in identifying the cuttlefish species. This finding highlights the robustness and effectiveness of spectral analysis as a tool for species identification, even in complex spatial contexts. The findings emphasize the potential of NIRS as a valuable tool in the field of fishery product authentication, offering a rapid and eco-friendly approach to species identification in the post-processing stages.

## 1. Introduction

The genus Sepia, as classified by Linnaeus in 1758, represents a group of considerable commercial importance, encompassing approximately 100 species [1]. This genus stands as the largest among the three genera identified within the *Sepiidae* family, as established by Leach in 1817. Among the European cuttlefish species, *Sepia officinalis* holds a significant position as the prevailing genus in the Mediterranean Sea (FAO fishing area 37). Nevertheless, its presence extends further to encompass the Eastern North and Central Atlantic Oceans (FAO fishing areas 27 and 34, respectively) [2]. 

Accurate species identification, especially in the fishery sector, is critical for ensuring food safety by detecting harmful toxins (i.e., domoic acid) or parasites (i.e., *Dicyemid* parasite) that may be present in certain cephalopods species [3,4]. Dichotomous keys serve as valuable tools utilized by scientists and professionals in the food industry to enable rigorous classification and taxonomy, ensuring accuracy and reliability in species identification for scientific investigations and commercial applications alike. Species identification is generally conducted on unprocessed products by an official authority and food business operator through visual analysis by examining the morphological characteristics of cuttlefish, i.e., coat color, corneal membrane, horny beak, and siphon structure. The anatomical complexity of these structures necessitates highly skilled personnel and specialized techniques to ensure accurate identification [5]. However, current identification techniques have notable limitations. In particular, the taxonomic approach is not applicable when morphological characteristics have been removed (i.e., cuttlebone, statoliths, beaks, and radula [6]), and the dichotomous keys employment are difficult to consider due to inconsistent description of the catalog proposed. However, molecular analysis for identification purposes overcomes these limitations applying to whole or prepared products; indeed, despite the significant accuracy provided by molecular methods, it is crucial to acknowledge that these techniques often rely on a limited sample size. This is primarily attributed to the resource-intensive nature of molecular analyses, which require specific reagents, laboratory infrastructure, and skilled personnel, making them expensive and time-consuming processes [7]. Additionally, the primary DNA-based methods utilized, such as PCR and DNA sequencing, due to the long preparation step of the sample, are not conducive to rapid and cost-effective species identification in field settings. 

A strategic, economic, non-destructive, rapid, and easy alternative approach applicable in every phase of the supply chain can be represented by the Near-InfraRed Spectroscopy (NIRS) technology for food control purposes [8,9,10,11]. Indeed, the Commission Implementing Regulation (EU) 2022/2503 has recently recognized the value of untargeted analysis (NIRS/UV-VIS) as a supporting device in assessing the physical status (fresh/frozen–thawed) of fishery products during official control [12]. The implementation of a fast and suitable technique to identify fishery species meets the need of food regulatory demand to comply with the labeling laws but also to protect the interest of the seafood business operator and consumers, guaranteeing the truthfulness of the supply chain in seafood products without the sample destruction [13]. To the best of our knowledge, studies on species identification using InfraRed Spectroscopy have primarily concentrated within the Teleost infraclass, and there is a lack of research specifically targeting cephalopod species identification using handheld-portable Near-Infrared Spectroscopy devices. In light of this, the present study aims to address this gap by focusing on four distinct cuttlefish species. Therefore, the primary aim is to assess the significant potential of Near-Infrared Spectroscopy (NIRS) in precisely identifying these prepared cuttlefish species at the initial stages of the complex supply chain.

## 2. Materials and Methods

### 2.1. Cuttlefish Sampling and Species Identification

A total of 93 individual frozen–thawed cuttlefishes of 4 species were collected in June 2022 from two wholesale fish plants in Chioggia (Venice, Italia). The species identification was performed prior to product processing as part of the company’s self-monitoring process. The procedure involved evaluating the morphological features of the cuttlefish species, which were obtained prior to the sample preparation methods, adhering to the standardized protocols of the company. The species considered included *S. officinalis* (3 batches; *n* = 31 and *n* = 9 samples fished in 27.7 and in 34 FAO fishing areas, respectively), *S. bertheloti* (1 batch; *n* = 9 samples fished in 34 FAO fishing areas), *S. aculeata* (1 batch; *n* = 10 samples fished in the 51 FAO fishing area), and *Sepiella inermis* (2 batches; *n* = 19 and *n* = 15 samples fished in the 57 and 71 FAO fishing areas, respectively). 

### 2.2. NIR Data Collection

The whole and refrigerated cuttlefish samples were subjected to NIRS measurement after the company’s standard procedures, which involved removing the skin, gut, and bones and storing the samples on ice. A PoliSPEC NIR (ITPhotonics in Breganze, Italy) portable spectrophotometer was used to obtain spectral data from each sample through a round scanning window of 3.2 cm^2^ placed in direct contact with the surface of the sample and scanning along the mantle. The spectral range scanned was from 900–1680 nm with a resolution of 2 nm. The individual spectrum of each cuttlefish was obtained by averaging the scans collected continuously for 5 s. The spectral data were recorded in reflectance units (R) and subsequently converted to absorbance units using the poliDATA 3.0.1 software (ITPhotonics in Breganze, Italy) by taking the logarithm of the reciprocal of R.

### 2.3. Identification Species Model

In the study, spectral data from cuttlefishes were analyzed in two forms: untreated and treated using different methods, namely standard normal variate (SNV), Savitzky–Golay, and derivative (none, first, and second). The investigation aimed to evaluate the best results and assess the effects of these treatments on the cuttlefish spectral data. The NIRS spectral data were processed using R software version 4.0.2 (R Core Team, 2020) for both unsupervised and supervised methods. For the unsupervised analysis, Principal Component Analysis (PCA) was employed as a descriptive tool to visualize the data distribution. On the other hand, the supervised approach involved utilizing Support Vector Machine (SVM), as reported in the works of Currò et al. [8,14]. Concisely, the SVM model was developed using the ‘caret’ package, employing both the svmLinear and svmRadial kernels. The training dataset underwent repeated hold-out-validation. For multi-class classification (classes > 3), the ‘one-against-one’ approach was utilized, entailing training k (k − 1)/2 binary classifiers and determining the appropriate class through a voting scheme. To improve the SVM performance, a grid search was conducted to fine-tune the C-value (Cost) in the Linear classifier and the Radial Basis Function sigma. This process involved exploring different combinations of these parameters to identify the most suitable model configuration. The SVM was employed in the development of the calibration model to assess the NIRS classification potential for species identification. Briefly, the complete dataset was divided into two sets: a training set and a testing set. The training set, which accounted for 70% of the samples (*n* = 66), was used to develop discrimination models. The testing set, representing 30% of the samples (*n* = 27), was employed to evaluate and validate the developed model. To validate the model, a hold-out validation approach was utilized. The dataset was split again, with 70% allocated to the training set for repeated cross-validation (with 10 settings and 5 repeats) and the remaining 30% forming the testing set. Table 1 provides a comprehensive breakdown of all sampled cuttlefish, along with their distribution into training and testing sets, including their respective varieties and the FAO fishing area of origin.

## 3. Results and Discussion

Species substitution in the fish industry is a widespread issue, particularly aggravated in post-processing stages at retailers and supermarkets. The deliberate substitution of high-value species with lower-quality alternatives is the most commonly observed form of fish fraud [15]. However, accidental substitution can also occur when species closely resemble each other, leading to mistaken identities. This practice primarily affects processed products and fillets that are challenging to identify using traditional morphological analysis. The inherent complexities in species identification significantly contribute to the prevalence of this problem, especially in retail settings. 

Among the results observed using untreated and treated data, the best performances were obtained for raw spectral data and were described in the following sections.

### 3.1. Exploratory Results

**Figure 1:**
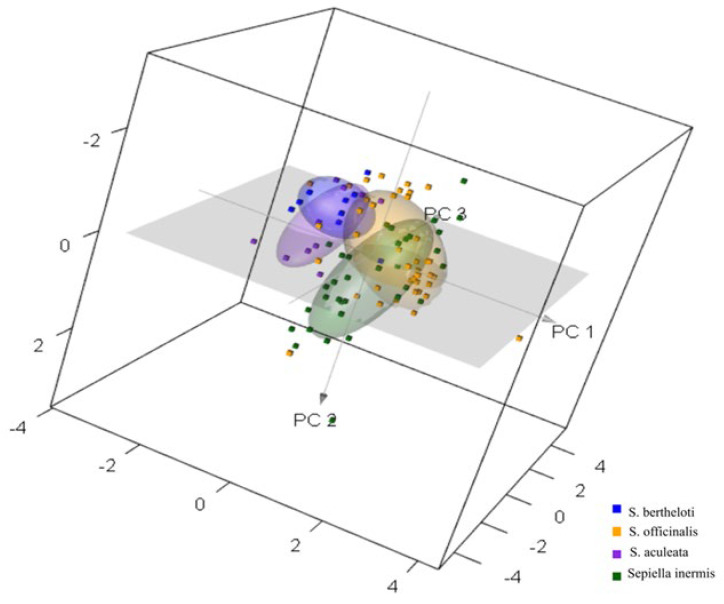
Principal component score plot for PC1, PC2, and PC3 of raw spectra.

Principal Component Analysis (PCA) was employed as an unsupervised method to qualitatively visualize differences among cuttlefish samples related to the different species offering a straightforward means of identifying potential clusters of samples [16]. In particular, Figure 1 depicts the score plots of the first three PCs of the groups of cuttlefish species. Notably, PC1, PC2, and PC3 explained 68%, 28%, and 3% of the total variance.

The high variance explained by PC1 indicates that it effectively captures the significant and relevant characteristics inherent in the raw spectrum. This emphasizes the importance of PC1 in representing the primary sources of variation among the samples. This observation implies that species identification could potentially be facilitated by leveraging the distinct spectral attributes present in the samples. However, a partial separation among samples related to the different cuttlefish species was observed, suggesting that species identification could be possible due to the different spectral attributes of the samples associated with the characteristics of the species considered. Nonetheless, it is important to acknowledge that while PCA is valuable for dimensionality reduction and highlighting major sources of variance, it may not capture all intricacies related to species classification, thus explaining the partial overlapping among groups.

The findings align with the outcomes of previous studies conducted by Ottavian et al. [17] and Lv et al. [18], where PCA was used for classifying fish species. These studies demonstrated that PCA groups’ segregation provided promising results for species classification; thus, the congruence between these studies and the current research suggests that PCA is a suitable method for exploring data trends in species differentiation.

**Figure 2:**
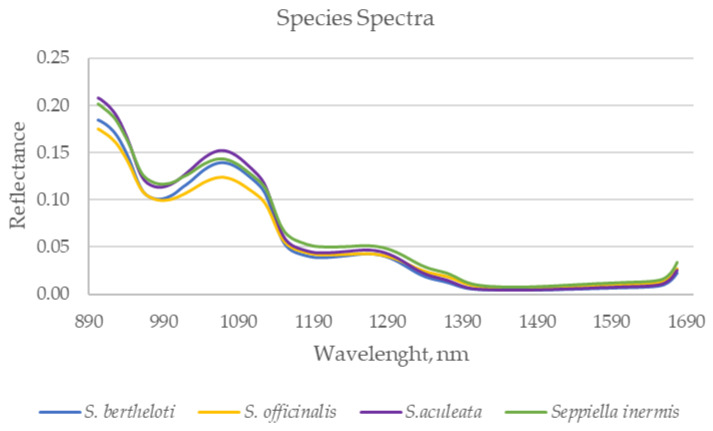
NIR spectra (mean) of cuttlefish species collected using the NIR portable device.

Figure 2 illustrates the average raw spectra acquired from cuttlefish species within the specified range (900–1680 nm). The observed divergences between species can likely be attributed to variations in their physical and chemical properties, which, in turn, are influenced by differences in their habitats and diets [14]. These environmental factors play a crucial role in shaping the spectrum of each species showing a noticeable graphical divergence among the species.

### 3.2. Classification by Species 

In recent years, there has been an exploration of the application of NIRS to recognize different fishery species [16,19]. However, to date, research on species identification utilizing NIRS has predominantly focused on species within the Teleost infraclass. In detail, the study of Cavallini et al. [10] focused on differentiating between two closely resembling flatfish species, *Synaptura cadenati* (Guinean sole; *n* = 50) and *Pleuronectes platessa* (European plaice; *n* = 50). The fillets of these species were analyzed using portable NIRS devices operating at wavelengths of 740–1070 nm and 908–1676 nm, as well as a benchtop NIRS device operating at wavelengths of 800–2500 nm. The collected spectra were then subjected to chemometric analysis employing Partial Least Squares-Discriminant Analysis (PLS-DA) classification model. Such a study demonstrated an accuracy of 94.1% in species classification using the portable NIRS device and an accuracy of 90.1% using the benchtop device. In the study conducted by Ottavian et al. [17], fish minced samples were classified according to the species considered (*Sparus aurata*, *n* = 106; *Mullus barbatus*, *n* = 106; *Solea vulgaris*, *n* = 88; *Xiphias gladius*, *n* = 175) using a NIRS reflectance device and a PLS-DA classification model. The samples were categorized, obtaining a classification accuracy of 100% in validation.

The study of Lv et al. [18], using an NIRS reflectance device (1000–1799 nm) and chemometrics (PCA-Linear Discriminant Analysis) to distinguish among seven different carp species (*Hypophthalmichthys molitrix*, *n* = 100; *Mylopharyngodon piceus*, *n* = 100; *Aristichthys nobilis*, *n* =100; *Ctenopharyngodon idellus*, *n* = 100; *Cyprinus carpio*, n = 80; *Carassius auratus*, *n* = 100; *Parabramis pekinensis*, *n* = 70), showed a complete classification capability (100%). Analog outcomes were noted in the investigation conducted by Alamprese et al. [20], wherein *Mullus surmuletus* (Red mullet) and *Pseudupeneus prayensis* (Atlantic mullet) were successfully identified using FT-NIR through the application of the Soft Independent Modelling of Class Analogies (SIMCA) technique. In contrast, the study conducted by Cozzolino et al. [21] demonstrated a lower classification capability compared to previous studies, probably because it was performed on by-products using an NIRS device (1100–2500 nm) to collect spectra. The classification accuracy (using Linear Discriminant Analysis) achieved using fish meals from *Salmon salar*, *Micromesistius poutassau*, and other species such as *Scomber scombrus* and *Clupea harengus* ranged from 70% to 90%. These studies demonstrate the potential of NIRS combined with chemometric analysis for species identification and classification in various fish species. The accuracy levels achieved vary across the studies, which can be influenced by factors such as the type of fish species, the specific NIRS device used, the wavelength range, and the analytical techniques employed. However, to the best of our knowledge, there is a noticeable gap in studies that specifically explore cephalopod species identification.

Indeed, to address the existing research gap, the present study focuses on four distinct cuttlefish species, including *Sepia officinalis* and *Sepia bertheloti*, which have overlapping distributions in the Central and Eastern Atlantic (FAO fishing area 34). The study aimed to assess the Near-Infrared Spectroscopy (NIRS) classification capability in differentiating and recognizing these cuttlefish species. From the obtained confusion matrix (Table 2), the overall accuracy of the NIRS classification was found to be 93%. 

The balanced accuracy, calculated for each class, demonstrated complete accuracy for the *S. bertheloti* and *Sepiella inermis* species. However, it was lower (93%) for *S. officinalis* and the lowest (67%) for *S. aculeata* species. Among the 27 samples in the test set, only 7% of cuttlefish were misclassified. The SVM linear classification model used in this study exhibited a perfect sensitivity (100%) in detecting positive cases for *S. bertheloti*, *S. officinalis*, and *Sepiella inermis*. This indicates that the model accurately identified all instances of these species in the dataset, reflecting a high level of accuracy in species detection. These results provide encouraging prospects for species identification and classification tasks using NIRS technology. However, the model showed lower sensitivity in identifying *S. aculeata* species, with two out of three samples being misclassified as *S. officinalis*. This misclassification affected the specificity of *S. officinalis* (86%). Nevertheless, the model demonstrated high specificity in correctly identifying negative cases for *S. bertheloti*, *S. aculeata*, and *Sepiella inermis* species. In the present study, there were four species considered, and two of them (*S. bertheloti* and *S. officinalis*) overlapped for the 34 FAO fishing area collections (Central Atlantic Oceans). However, this overlap did not have any negative impact on species identification. Similar to the study conducted by Varrà et al. [22], the present study confirmed that the samples exhibited different spectral attributes associated with the characteristics of each species. Indeed, despite sharing the same fishing area, the distinct spectral properties allowed for accurate identification and differentiation between *S. bertheloti* and *S. officinalis*. This finding emphasizes that even when species overlap spatially (habitat), their unique spectral attributes remain reliable markers for species identification and are more prominently identified through the supervised approach. In detail, with NIRS being an untargeted approach, the combination of essential molecular vibrations and overtones associated with specific functional chemical groups highlight the capability of NIRS to classify cuttlefish based on species as a qualitative trait. Specifically, this differentiation is attributed to the evaluation of the molecular phenotype derived from averaging the vibration modes of all molecules within the specimen. The molecular phenotype exhibits variations among species within the same genus, reflecting their distinct genome expression [23,24].

## 4. Conclusions

This study highlights the efficacy of NIRS classification in identifying the four species of cuttlefish examined. Despite an overall high accuracy of 93% in species identification, it is imperative to account for greater sample variability within each species to enhance the precision of the classification process. Nonetheless, the findings underscore the potential of rapid, eco-friendly, and easy-to-use NIRS devices for the online authentication of cuttlefish species, especially when morphological characteristics have been removed and the dichotomous keys employment are difficult to be considered (prepared product). However, the practical implications of these results are substantial; indeed, the ability to swiftly analyze a large number of samples not only strengthens consumer protection against adulteration and fraudulent claims but also empowers commercial stakeholders to verify the trustworthiness of their suppliers and ensure the integrity of received prepared products. Furthermore, this approach provides a legitimate means to investigate suspected fraudulent activities, enabling the evaluation and substantiation of such claims through more sophisticated analyses. This study contributes to highlighting the potential of NIRS-based species identification as a valuable tool for quality control and supply chain integrity in the context of fishery product authentication.

## Figures and Tables

**Table 1:** Sample description according to the training and testing sets used in the hold-out validation.

	n. of Samples	n. Per Species	FAO Fishing Area
Training	66	*S. bertheloti, n.* 7	34
		*S. officinalis, n.* 28	27.7 (*n.* 22); 34 (*n.* 6)
		*S. aculeata, n.* 7	51
		*Sepiella inermis*, *n*. 24	57 (*n*. 13), 71 (*n*. 11)
Testing	27	*S. bertheloti, n.* 2	34
		*S. officinalis, n.* 12	27.7 (*n.* 9); 34 (*n.* 3)
		*S. aculeata, n.* 3	51
		*Sepiella inermis*, *n*. 10	57 (*n*. 6); 71 (*n*. 4)

**Table 2:** Performance of classification of Support Vector Machine model to discriminate cuttlefish according to the species in hold-out validation.

	Reference Species			
Predicted Species	*S. bertheloti*	*S. officinalis*	*S. aculeata*	*Seppiella inermis*
*S. bertheloti*	2	0	0	0
*S. officinalis*	0	12	2	0
*S. aculeata*	0	0	1	0
*Seppiella inermis*	0	0	0	10
Overall Accuracy (%)	93			
Balanced Accuracy (%)	100	93	66	100

## Data Availability

Not applicable.
